# NETO2 promotes esophageal cancer progression by inducing proliferation and metastasis via PI3K/AKT and ERK pathway

**DOI:** 10.7150/ijbs.53795

**Published:** 2021-01-01

**Authors:** Jia-cheng Xu, Tian-yin Chen, Le-tai Liao, Tao Chen, Quan-lin Li, Jia-xin Xu, Jian-wei Hu, Ping-hong Zhou, Yi-qun Zhang

**Affiliations:** 1 Endoscopy Center and Endoscopy Research Institute, Zhongshan Hospital, Fudan University, No. 180 FengLin Road, Shanghai 200032, China.; 2 Department of Emergency Surgery, First Affiliated Hospital of Nanchang University, Nanchang, China.; 3 Endoscopy Center, East Hospital, Tongji University, Shanghai, China.

**Keywords:** Esophageal squamous cell carcinoma, NETO2, Proliferation, Metastasis, PI3K/AKT and ERK

## Abstract

Esophageal squamous cell carcinoma (ESCC) causes aggressive and lethal malignancies with extremely poor prognoses, and accounts for about 90% of cases of esophageal cancer. Neuropilin and tolloid-like 2 (NETO2) protein coding genes have been associated with various human cancers. Nevertheless, little information is reported about the phenotypic expression and its clinical significance in ESCC progression. Here, our study found that NETO2 expression in ESCC patients was associated with tumor clinical stage and lymph node metastasis status. Gain-of-function and loss-of-function analyses showed that NETO2 stimulated ESCC cell proliferation while suppressing apoptosis *in vitro* and enhanced tumor growth *in vivo*. Moreover, knockdown of NETO2 significantly inhibited migration and invasion in combination with regulation of epithelial-mesenchymal transition (EMT) related markers. Mechanistically, overexpression of NETO2 increased the phosphorylation of ERK, PI3k/AKT, and Nuclear factor erythroid-2-related factor 2(Nrf2), whereas silencing NETO2 decreased the phosphorylation of these targets. Our data suggest that Nrf2 was a critical downstream event responsible for triggering the PI3K/AKT and ERK signaling pathways and plays a crucial role in NETO2-mediated tumorigenesis. Taken together, NETO2 acts as an oncogene and might serve as a novel therapeutic target or prognostic biomarker in ESCC patients.

## Introduction

Esophageal squamous-cell carcinoma (ESCC) is one of the most aggressive and lethal malignant tumors in China and some other parts of the world 1. Among stage IV patients, the medial survival time is less than one year. Further, among stage III patients, the survival rate is 10% over 5 years 2,3. Esophagostomy is the most comprehensive but complex and technically challenging surgical treatment for patients with ESCC 4. However, more than 20% of patients already have regional lymph node metastasis and advanced local disease 5, which is the major cause of ESCC-related death and leads to tumor lesion recurrence and metastasis 6. Recently, many researchers have determined the prognostic markers of proliferation and metastasis of ESCC such as PGK1, TBL1XR1, BRCA1 and identified a number of driver events 3,7,8. However, the prognosis for patients with ESCC is still poor. Therefore, elucidation the underlying molecular mechanisms of ESCC will allow for the development of developing more innovative and effective approaches to reduce the mortality of patients suffering from this disease.

NETO2 genes encode transmembrane proteins that belong to the CUB domain and LDLa-containing unique subfamily 9, which modulate the neuronal kainate receptors (KARs) by slowing or accelerating recovery from desensitization 10,11. In addition, NETO2 also able to interact with the K+/Cl- cotransporter (KCC2) by enhancing its recycling in hippocampal neurons 12. Although NETO2 was originally thought to be a neuron-specific protein 13, increasing evidence indicates NETO2 expression is also detectable in several types of cancers. A recent study found that NETO2 plays an important role in tumor progression in pancreatic and nasopharyngeal carcinomas 14,15. High levels of NETO2 were correlated with clinicopathologic features and poor survival in colorectal carcinomas 16. Moreover, NETO2 is directly correlated with risk of death in HCC patients 17. However, little is reported about NETO2's phenotypic expression pattern and its clinical significance in ESCC progression.

In the present study, we examined the clinical significance of NETO2 and further analyzed the correlation between high expression of NETO2 with poor prognosis and patient survival. Furthermore, NETO2 depletion inhibits cell proliferation, migration and invasion, while enhancing apoptosis in cells via the ERK and PI3K/AKT pathways. This study demonstrates that NETO2 might serve as an independent indicator for unfavorable prognoses in ESCC.

## Material and Methods

### Patients and specimens

This study was approved by Ethics Review Board of Zhongshan Hospital. 103 esophageal cancer samples and paired normal tissues (5cm away from the tumor margin) were obtained from patients who underwent Esophagostomy without chemotherapy or radiotherapy before surgery between November 2010 and September 2011 at Zhongshan Hospital, Fudan University for immunohistochemical assay. Patients were followed until January 2016 and informed consent was obtained from all patients.

### Cell culture and reagents

The human ESCC cell lines ECA109 KYSE150 and TE-1 cells were purchased from the cell bank of the Shanghai Institutes for Biological Sciences (Shanghai, China). The Kyse150 and TE1 cells were cultured in RPMI 1640 (Hyclone, Logan, TX, USA), and ECA109 was maintained in DEME (Gibco, Grand Island, NY, USA) in 37 °C with 5% CO2. All media were supplemented with 10% fetal bovine serum (Gibco, NY, USA) and 1% penicillin/streptomycin (Hyclone). For hypoxia treatment, cells were cultured in 1% O2, 5% CO2, and balanced with N2 gas (94%).

SCH772948 and LY294002 were purchased from MCE (New Jersey, USA). For Western blot analysis, ESCC cells were incubated with 20 μM SCH772948 and 25 μM LY294002 for 2 h. For functional assays, ESCC cells were treated with same concentration for 24 h.

### Cell transfection

Cells were seeded in six-well plates, and the medium was replaced with Opti-MEM. Small interfering RNA (siRNA; GenePharma, China) and Lipofectamine 2000 transfection reagent (Life Technologies, USA) were transfected into the cells according to the manufacturer's recommendations. NETO2 plasmids (Longqian Biotech) or control plasmids were transfected into cells by using Viafect Transfection Reagent (Promega). The target siRNA sequence list as follows: NETO2 siRNA-1: 5′-GCAGGAGUAUUUGAACAAA-3′; NETO2 siRNA-2 5′-GGUUCCUAGAUUAUCAAAU-3′; Nrf2 siRNA: siRNA-1 5′-CCAGAACACUCAGUGGAAUTT-3′; siRNA-2 5′-GACAGAAGUUGACAAUUAUTT-3′; siRNA-3 GGUUGAGACUACCAUGGUUTT-3′.

Lentiviruses that expressed NETO2-siRNA and were purchased from Genechem (Shanghai, China). Cells were harvested 48 h after transfection of lentivirus according to the manufacturer's protocol. Cells were analyzed for NETO2 protein expression using Western blot with analogous antibodies.

### Quantitative real-time PCR

Total RNA was extracted from cells or samples by using TRIzol reagent (Takara, Shiga, Japan). Real-time PCR was performed using SYBR-Green master mix (Takara) and products were detected by StepOne Plus system (Applied Biosystems, Foster City, CA). GAPDH was served as an endogenous control. The primers sequences are listed as follows: NETO2 forward: AGATGGGCCATTTGGTTTCTC; NETO2 reverse: TGCTCGAAATCCCAGTCCTTC; Nrf2 forward: TCAGCGACGGAAAGAGTATGA; Nrf2 Reverse: CCACTGGTTTCTGACTGGATGT.

### Western blot analysis

Cells were lysed in RIPA buffer (Cell Signaling, USA) and quantified using the BCA protein assay (Beyotime, Shanghai, China). Equal amounts of protein were subjected to electrophoresis on SDS-PAGE and were probed with the following primary antibodies: NETO2, Nrf2, p-Nrf2 (Ser40) purchased from Abcam. Caspase 3, cleaved caspase 3, Bcl-2, p-AKT (Thr308), AKT, p-mTOR, p-ERK1/2 (Thr202/Tyr204), ERK1/2, E-cadherin, N-cadherin, slug, and snail were purchased from Cell Signaling Technology (Danvers, MA, USA). The protein bands were measured using a Gel Doc 2000 (Bio-Rad).

### Cell proliferation and colony‐forming assays

Approximately 1000 ECA109 Kyse150 cells were plated in 96-well plates and cultured for 24 h. Cell viability was assessed using Cell Counting Kit-8 (Dojindo, Kumamoto, Japan) according to the manufacturer's instructions.

500 cells in log-phase were plated into 6-well plates. After a two-week incubation period, colonies were fixed with 4% paraformaldehyde for 30 min and stained with 0.1% crystal violet solution (Sigma, USA) for 15 min. Colonies (>50 cell) were counted under microscopy.

### Xenografted animals

BALB/c male nude mice, 4-6 weeks old, were purchased from the Shanghai SLAC Laboratory Animal Co., Ltd. (Shanghai, China). All animal experiments were conducted in Xinhua hospital Shanghai Jiao Tong University. ECA109 cells (Lv-NC, Lv-NETO2) were resuspended at a density of 2×10^6^ in phosphate-buffered saline and inoculated subcutaneously into the right axilla of the nude mice (5 mice/group). Tumor growth was monitored every week. On day 28, the mice were sacrificed, and the tumors were dissected and weighed. Tumor volume was measured using a digital caliper and calculated as 4 π/3 × length/2 × width^2^ (mm). All animal experiments were approved by the Animal Care Committee of Xinhua hospital Shanghai Jiao Tong University.

### Cell apoptosis assay

To perform cell apoptosis assays, ESCC cells were washed twice and resuspended with 100 μL 1×binding buffer containing 5 μl of AnnexinV-FITC and 5 μl of PI staining solution and incubated for 30 minutes in the dark. Then the samples were mixed into 400 μl 1×Binding Buffer and measured using flow cytometry (BD Biosciences, USA).

### Hoechst 33342 staining

Nuclear fragmentation was visualized by staining apoptotic nuclei with Hoechst 33342 (Invitrogen, Carlsbad, CA, USA). ESCC cells were collected and fixed with a 3:1 methanol/acetic ratio for 15 min at room temperature. The fixed cells were subsequently stained with 5 μg/mL Hoechst 33342 for 10 min. Images were captured using a fluorescence microscope (Leica, Wetzlar, Germany).

### Migration and invasion assays

Invasion was assessed using 24-well Matrigel Invasion Chambers (Corning, NY, USA). ECA109 (5×10^4^) and Kyse150 cells (7×10^4^) were added to coated filters in serum-free medium. Media containing 20% fetal bovine serum (FBS) was added to the lower chamber as a chemo-attractant. After 36 hours at 37 °C in a 5% CO2 basal incubator, cells were fixed with 4% paraformaldehyde, stained with 0.1% crystal violet solution, and photographed. The migration assays were conducted used 5×10^4^ ECA109 and Kyse150 cells for 24 h and 36 h incubation periods, respectively, in a similar fashion using 8µm transwell filters without coating with Matrigel (Corning).

### Immunohistochemistry

We performed Immunohistochemical staining following a standard immunoperoxidase staining procedure. Tissues were stored in 4% paraformaldehyde and then embedded in paraffin, cut into 5 mm sections, and mounted on slides. The percentage of NETO2 was scored on a scale 0-4 (no staining score=0, < 25% score=1, 26-50% score=2, 51-75% score=3, 75-100% score=4). Additionally, the NETO2 staining intensity was scored as 0 (negative), 1 (weak), 2 (moderate), 3 (strong). The final score was defined as the sum of these parameters.

### Statistical analyses

All experiments were performed at least three times. Statistical analyses were performed using Student's *t* test with GraphPad Prism, Pearson chi-square test and Fisher's Exact test with SPSS software. Kaplan-Meier analysis was used for the survival analysis. All quantified data are expressed as the mean ± standard deviation, unless otherwise stated. *P* values < 0.05 were considered to be significant. Significant differences are indicated *P* < 0.01.

## Results

### NETO2 is aberrantly expressed in ESCC tissues and positively correlated with poor prognosis in ESCC patients

**Figure 1:**
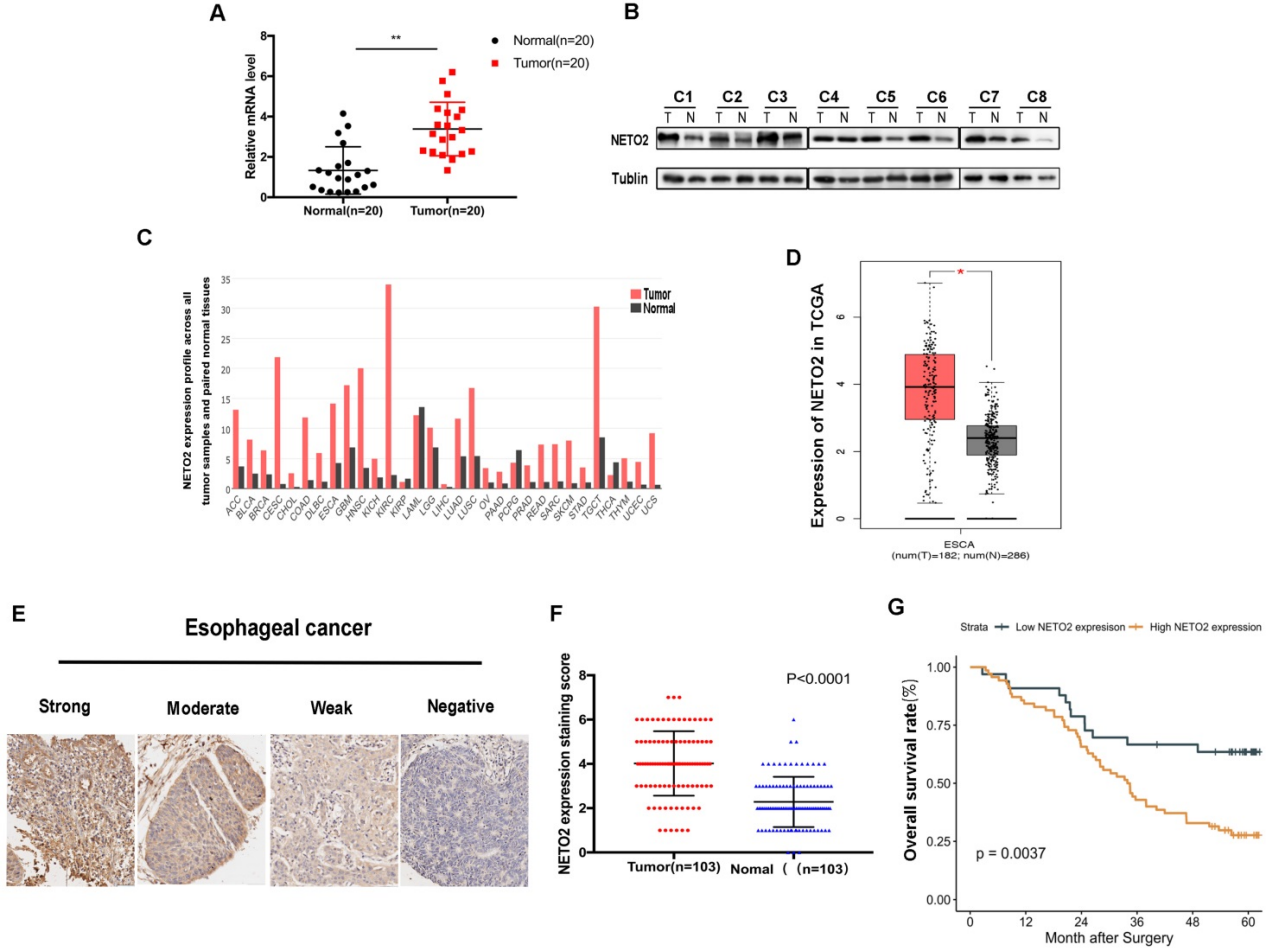
NETO2 is highly expressed and associated with progression and poor prognosis of ESCC patients. **A,** NETO2 mRNA levels were significantly higher in ESCC compared with paired non-tumor tissues. **B,** Protein expression levels of NETO2 in eight pairs of ESCC tissues. **C,** mRNA level of NETO2 in all tumor samples and paired normal tissues (TCGA). **D,** mRNA level of NETO2 in ESCC and normal tissues (TCGA). **E,** Representative images of weak, moderate, strong and nagative NETO2 IHC staining. **F,** Scatterplots of the average staining scores of NETO2 expression in ESCC and normal tissues. **G,** Kaplan-Meier overall survival curve of ESCC patients correlated with NETO2 expression. *P < 0.05, **P < 0.01.

To explore the phenotypic expression and clinical significance of NETO2 in ESCC progression, we first examined the mRNA and protein level of NETO2 in cancerous specimens compared with paired normal tissues (Figure 1A,B). The results of RT-PCR and western blot showed that NETO2 are predominantly expression in ESCC specimens which is supported by TCGA database (Figure 1D). Among TCGA database, esophageal carcinoma is one of the highest expression level of NETO2 (Figure 1C). Furthermore, the correlation between NETO2 overexpression and clinicopathologic features in ESCC patients was evaluated by IHC assay (Figure 1E). As shown in Table 1, NETO2 overexpression was associated with ESCC Histological grade (*P*=0.005), TNM stage (*P*=0.003), Tumor size (*P*=0.024), depth of invasion (*P*<0.01) and lymph node metastasis (*P*=0.007) but we did not observe difference with patient's age and sex. Kaplan-Meier analysis showed that patients in NETO2-high group have lower OS than low NETO2-expression patients (Figure 1G). Our study indicated that NETO2 was upregulated and plays a prominent role in ESCC progression.

### NETO2 promotes ESCC cell proliferation abilities both *in vitro* and *in vivo*

**Figure 2:**
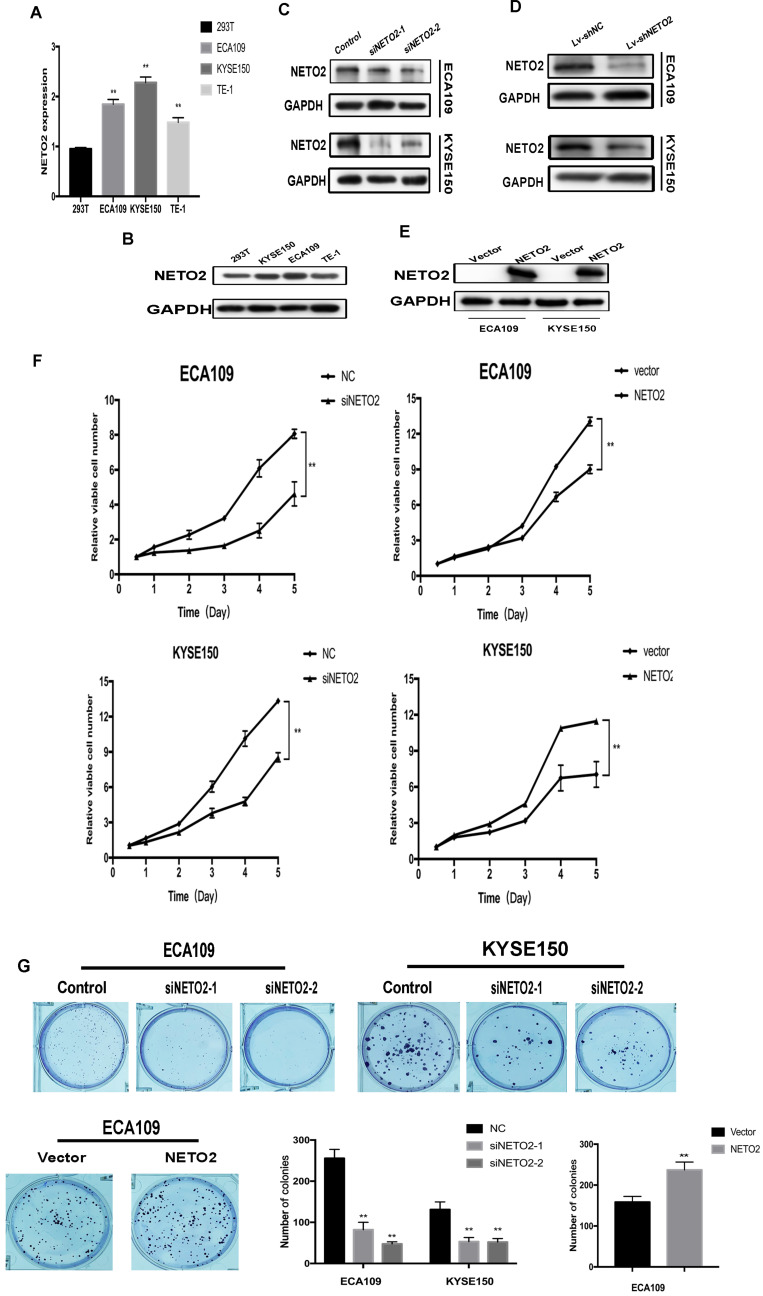
NETO2 promotes ESCC cell proliferation and viability. **A and B,** NETO2 expression in 293T and ESCC cell lines. **C,** NETO2 expression in ECA109 and KYSE150 cell lines transfected with NC and siNETO. **D**, Knockdown on NETO2 protein levels in ESCC cells infected with shNETO2 lentivirus. **E**, Effects of NETO2 overexpression in ECA109 and KYSE150 cells. **F and G,** CCK-8 assays and plate colony formation assays were conducted to determine the function of NETO2 on proliferation capabilities. All data are presented as mean ± SD and all the experiments were repeated 3 times. Significant differences are indicated by *P < 0.05, **P < 0.01.

To evaluate the ability of NETO2 on ESCC cell function, we first evaluated the endogenous NETO2 expression in 293T cells and three ESCC cell lines (ECA109, KYSE150 and TE-1). Because of no normal esophageal cells were incubated, we used 293T cells as negative control 18. ESCC cell lines showed higher NETO2 expression than 293T cells (Figure 2A,B). Then, we both downregulated and overexpressed NETO2 in ECA109 and KYSE150 cells, and the results were evaluated with qRT-PCR and western blot assays (Figure 2C,E).

CCK-8 assays and plate colony formation assays were conducted to determine the function of NETO2 on proliferation capabilities. In Figure 2F,G, transfection of the NETO2-siRNA obviously suppressed ESCC cell proliferation compared with mock cells, while overexpression NETO2 enhanced growth abilities. Collectively, our findings indicate that NETO2 stimulates ESCC cell proliferation.

**Figure 3:**
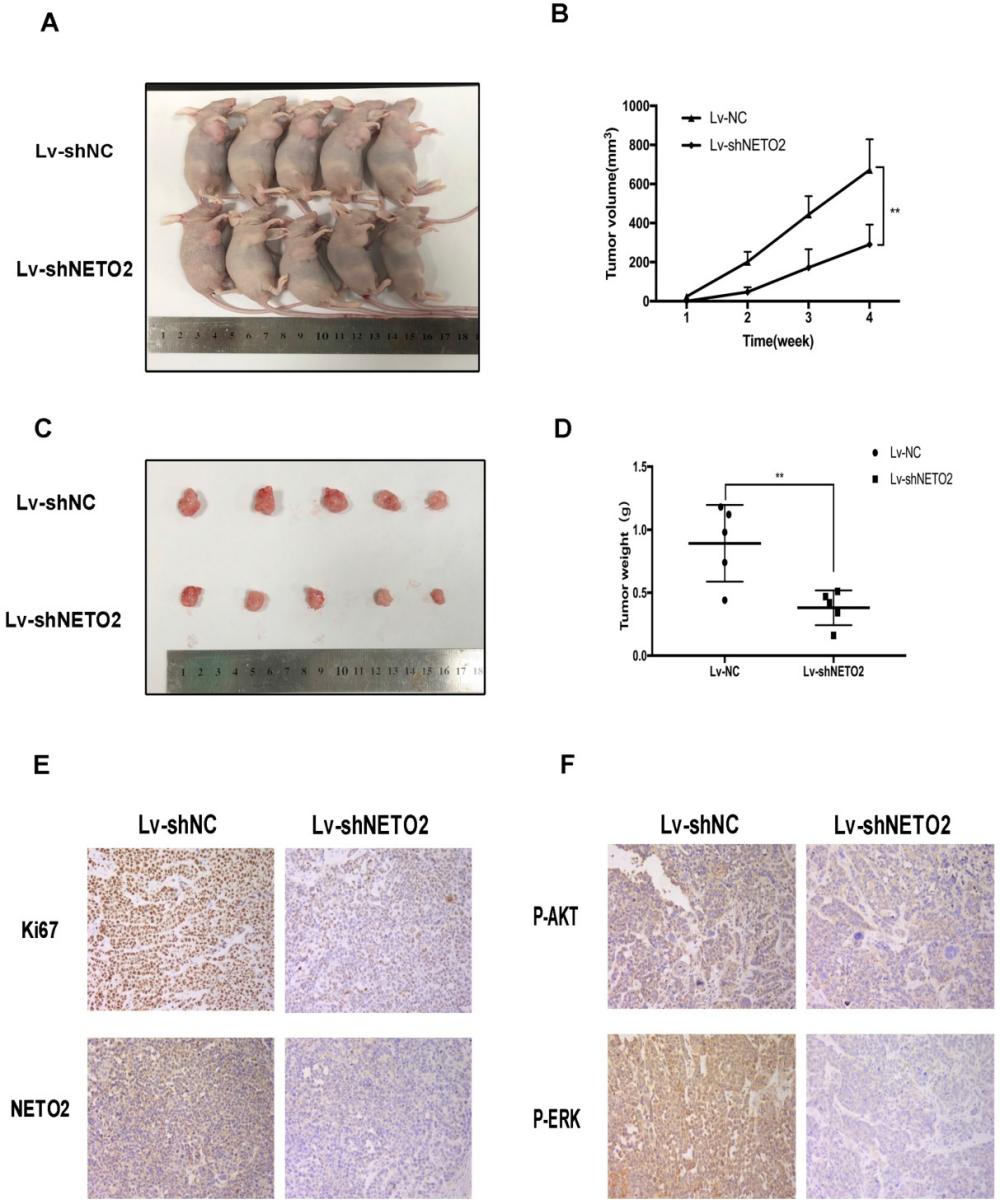
NETO2 silencing retards ECA109 cells tumorigenesis *in vivo*. **A-D,** Images of 5 representative mice from Lv-shNC/Lv-shNETO2 group are shown. Tumor sizes and tumor weights were measured. **E and F,** The expression of c Ki67, p-AKT and p-ERK were evaluated by IHC analysis in xenograft tumor; scale bar = 100 µm. All data are presented as mean ± SD and all the experiments were repeated 3 times. Significant differences are indicated by *P < 0.05, **P < 0.01.

Additionally, to further investigate the role of NETO2 in tumor progression, we established ECA109 cell lines with specific NETO2 shRNA stably downregulating NETO2 (Figure 2C). Next, we subcutaneously transplanted ECA109 cells into nude mice. Tumor volumes of NETO2-depleted xenograft were smaller than that seen in control mice (Figure 3A,B). Tumor weights were also lower in the NETO2-knowndown group (Figure 3C,D). IHC staining of the proliferative marker Ki67 were lower in NETO2 shRNA tumors, which is consistent with our data *in vitro* (Figure 3E,F).

### Silencing of NETO2 induces ESCC cell apoptosis

**Figure 4:**
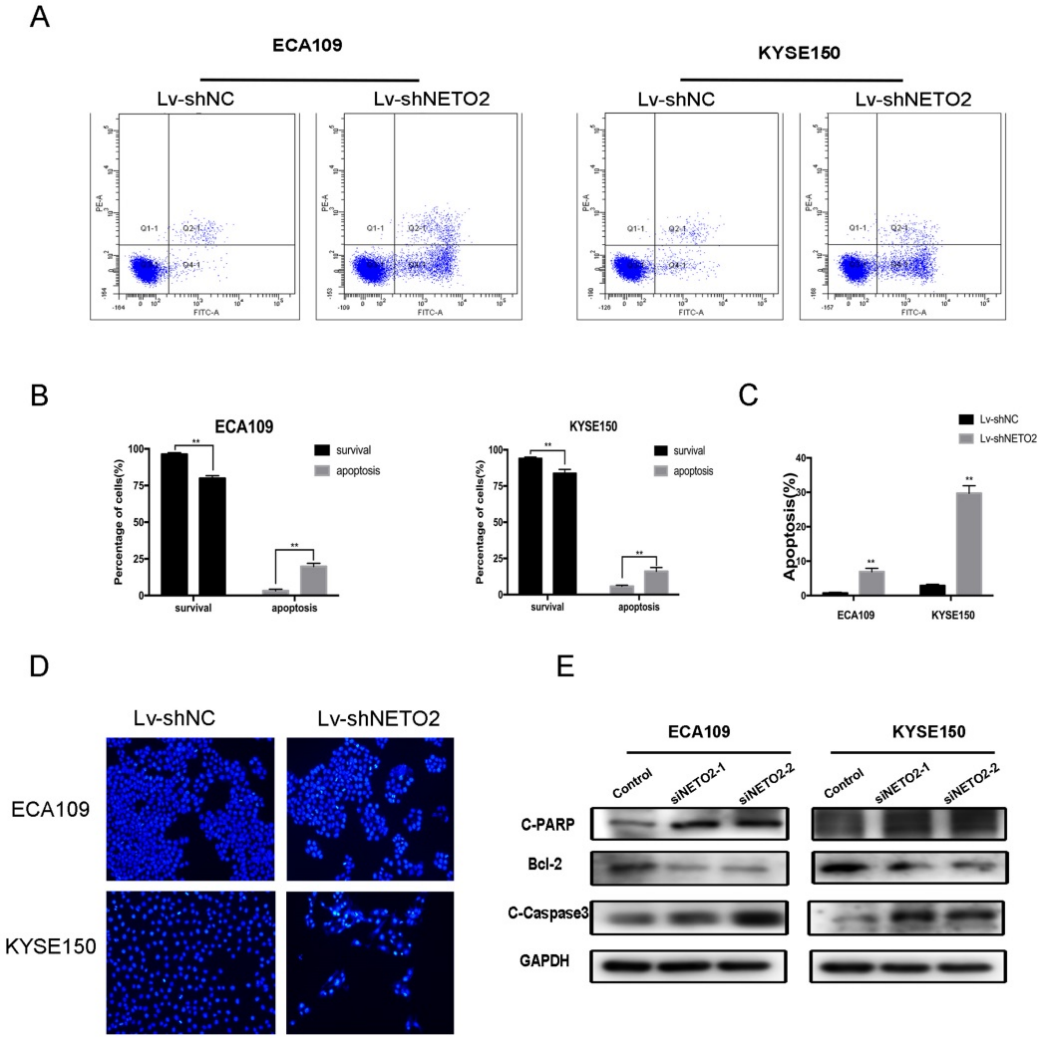
Apoptosis of ESCC cells after NETO2-knockdown. **A,** Apoptosis was assessed by flow cytometry with annexin V-FITC/propidium iodide (PI) staining. The x-axis represents annexinV-FITC, and the y-axis represents propidium iodide (PI) staining. **B,** There was a significant difference of apoptosis rate between Lv-shNC and Lv-shNETO2 groups. **C and D,** Nuclear morphological changes associated with apoptosis were evaluated by Hoechst 33342 staining. **E.** NETO2-knockdown increased C-PARP, C-caspase3 but suppressed Bcl-2 expression and the indicated factors was examined by western blot. GAPDH was used as the loading control. All data are presented as mean ± SD and all the experiments were repeated 3 times. Significant differences are indicated by *P < 0.05, **P < 0.01.

To shed light on the potential mechanism underlying the role of NETO2 in ESCC cell progression, we performed an apoptosis assay with Annexin V/PI double staining and flow cytometry. The cell apoptosis assay showed that the proportions of apoptotic ESCC cells in the Lv-shNETO2 groups were higher than those in the Lv-shNC groups (Figure 4A,B). We also performed the Hoechst 33342 staining assay to calculate the apoptosis outcomes and found that NETO2 knockdown cells contained more chromatin condensation and apoptotic body formation (Figure 4C,D).

We also used western blot assays to examine the expression of apoptosis-related proteins. Silencing NETO2 dramatically increased activation of caspase3 and activation of PARP, but downregulated Bcl-2 expression (Figure 4E). Taken together, this data indicates that NETO2 is involved in cell death-related protein expression, thus inducing ESCC cell apoptosis.

### NETO2 promote ESCC cells migration and invasion via regulation of EMT-related proteins

**Figure 5:**
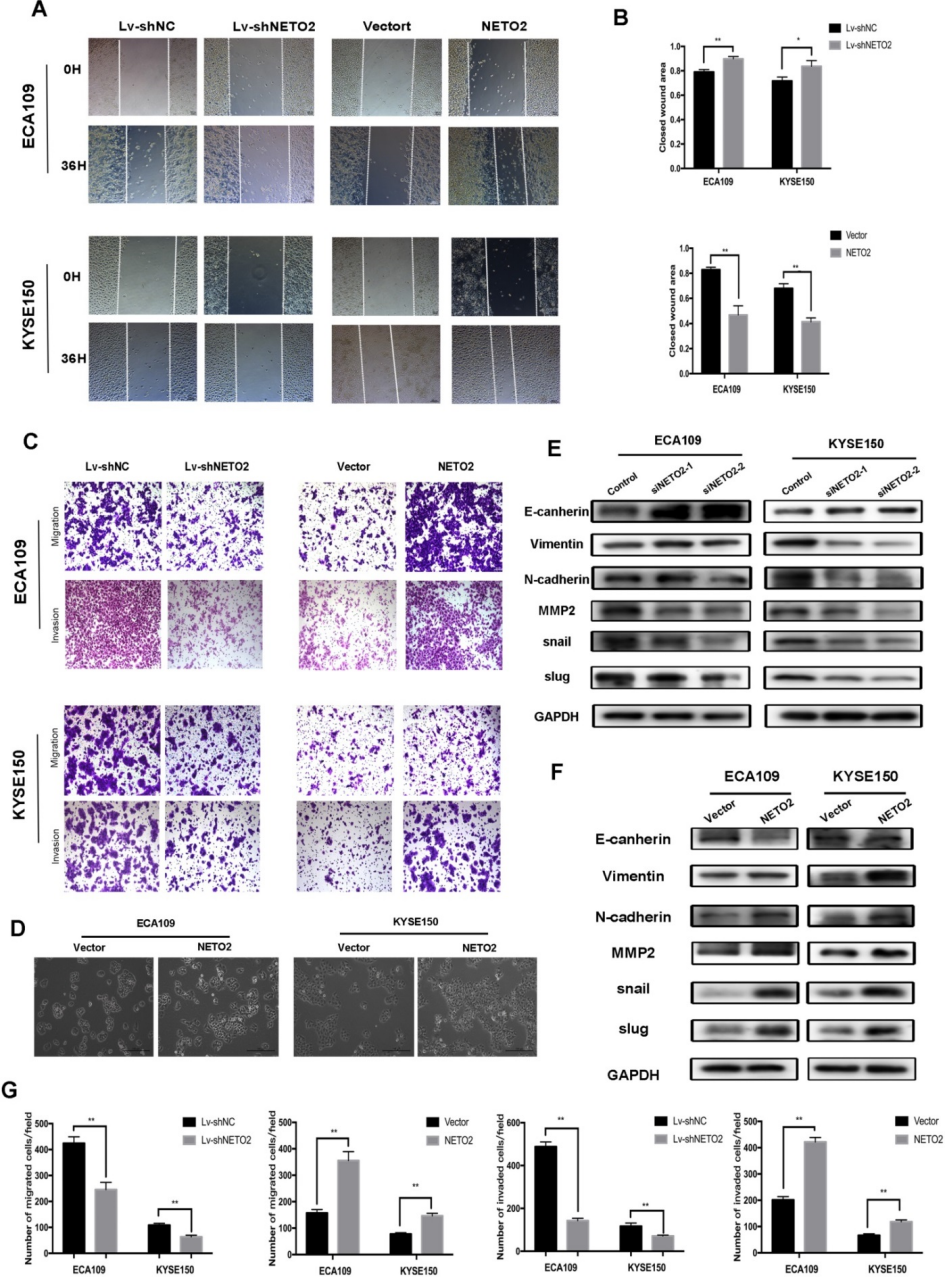
NETO2 promotes ESCC cell migration and invasion by inducing EMT. **A and B,** Wound healing assay was used to examine cell migration in ECA109 and KYSE150 cells transfected with Lv-shNETO2 or the NETO2 overexpression plasmid. **C,** Transwell assays were used to analyze ESCC cells motility. **D,** Morphologies of ECA109 and KYSE150 cells were examined under a microscope. **E and F,** Western blot analysis of E-cadherin, N-cadherin and snail slug and MMP2 levels in ESCC cells transfected with siNETO2 or the NETO2 overexpression plasmid. **G,** Effects of NETO2 knockdown and overexpression on ESCC cells motility. All data are presented as mean ± SD and all the experiments were repeated 3 times. Significant differences are indicated by *P < 0.05, **P < 0.01.

Our clinicopathologic association findings revealed that higher levels of NETO2 were associated with lymph node metastasis. To investigate whether NETO2 might be involved in the migration in ESCC cells, wound migration assays and transwell assays were performed. Knockdown NETO2 significantly suppressed the migration capabilities, while overexpression of NETO2 markedly enhanced these abilities as compared to the negative control group (Figure 5C,G). Similar results were also seen in the monolayer wound-healing assay (Figure 5A,B). The morphology of NETO2-forced expression cells yielded multi-angle shapes or shuttle shapes, and the multiple apophysis yielded irregular shapes (Figure 5D). In contrast, NETO2-knockdown led to negligible effect in its formation. Taken together, these results suggested that NETO2 enhanced the metastasis of ESCC cells and further explained the aforementioned function of NETO2 in the advanced clinical stage and metastasis of ESCC patients.

Furthermore, Western blot analysis was used to detect the expression of EMT markers to determine whether EMT was involved in metastasis of cancer cells. Depletion of NETO2 promoted the protein expression of E-cadherin and suppressed N-cadherin, snail, slug and MMP2 (Figure 5E). Overexpression of NETO2 led to the opposite effects (Figure 5F). Thus, these results indicated NETO2 promotes ESCC cells migration and invasion via regulation of EMT markers.

### NETO2 activates ERK and PI3K/AKT pathway

**Figure 6:**
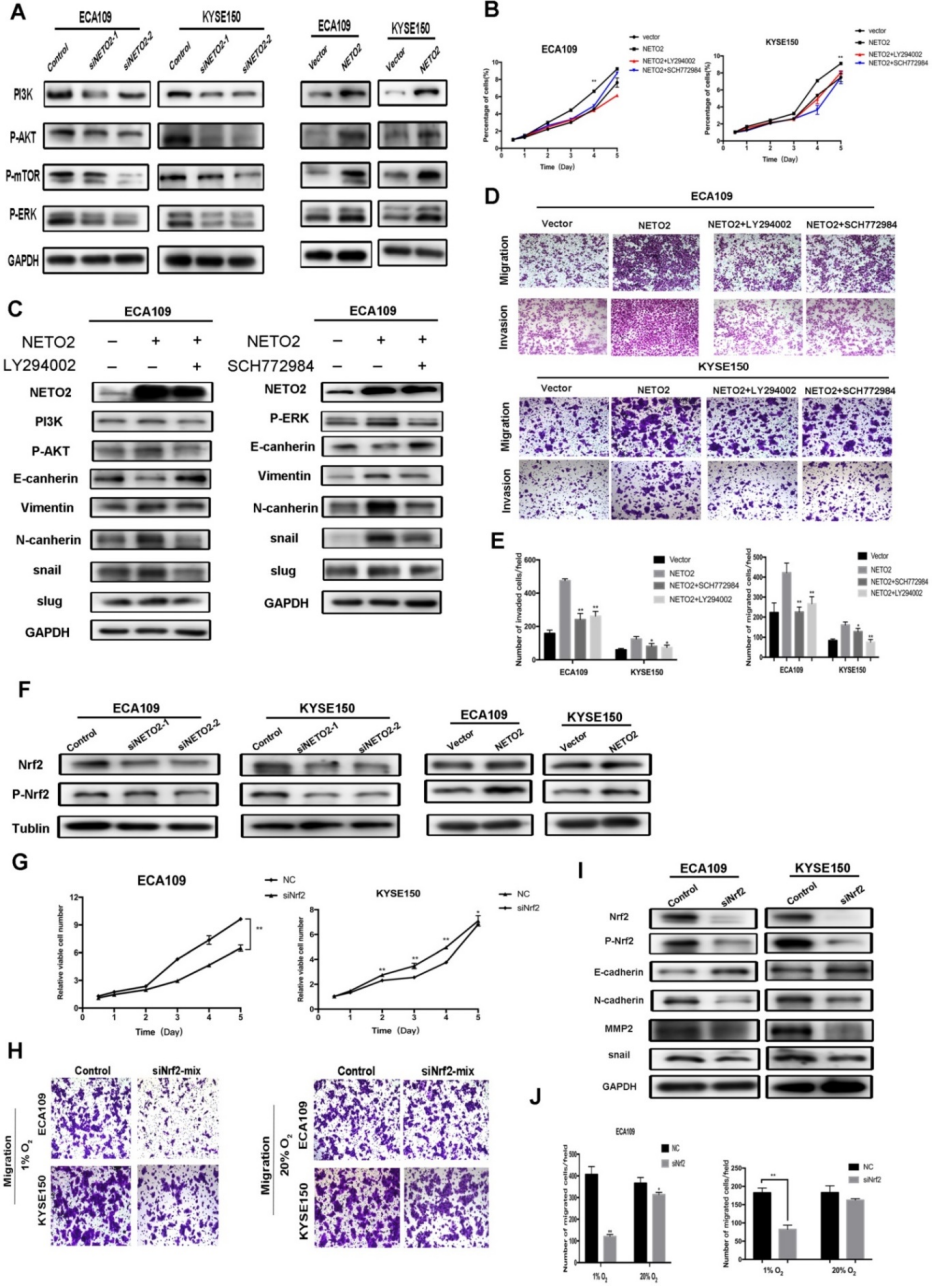
Inhibition of PI3K and ERK antagonizes NETO2-mediated proliferation, migration and invasion of ESCC cells. Nrf2 is associated with NETO2 to influence ESCC cell tumorigenesis ability. **A,** Western blot analysis of PI3K, p-AKT and p-ERK expression in indicated cells. GAPDH was used as the loading control. **B,** CCK8 assay for NETO2 overexpressing and vector cells with or without inhibitor treatment. **C,** Western blot analysis of EMT-related proteins for NETO2- overexpressed cells with or without inhibitor treatment. **D and E,** Transwell assays were used to analyze ESCC cell migration and invasion ability. **F,** Western blot analysis of expression of Nrf2 and p-Nrf2 with NETO2 knockdown and overexpression in ESCC cells. **G,** Cell proliferation assay were conducted to analyze the effects of Nrf2 on ESCC cell proliferation ability. **H,** In the hypoxic microenvironment, knockdown of Nrf2 led to an obvious effect on cell migration and invasion compared with basal conditions. **I,** E-cadherin, N-cadherin, MMP2 and snail expression levels were analyzed by Western blot analysis in cells transfected with siNrf2-mix. GAPDH was used as a loading control. **J,** Number of migrated cells according to Transwell data. All data are presented as mean ± SD and all the experiments were repeated 3 times. Significant differences are indicated by *P < 0.05, **P < 0.01.

Since the MAPK/ERK kinase, (MEK)-ERK, and PI3K/AKT pathways play important roles in cancer cell proliferation and metastasis, we assessed the phosphorylation of ERK1/2 PI3K AKT in cells with knock down and overexpression of NETO2 (Figure 6A). Western blot results showed that loss of NETO2 function significantly decreased P-ERK1/2 and P-AKT expression which is consistent with our IHC staining assay *in vivo* (Figure 3F). In contrast, the gain of NETO2 function markedly increases phosphorylation of them. To clarify whether NETO2 contributed to malignant progress in ESCC cells through activation of ERK and PI3K/AKT pathways, we treated ESCC cells with an ERK inhibitor SCH772984 or a PI3K inhibitor LY294002. Treatment with the inhibitor partially abrogated NETO2 overexpression-induced proliferation and metastasis (Figure 6B, D-E). As expected, inhibitor treatment also reduced the expression levels of EMT-related proteins (Figure 6C). Therefore, NETO2 promotes ESCC cell growth and metastasis, at least partially, through activation of MAPK/ERK and PI3K/AKT signaling pathways.

### Nrf2 involved in NETO2-induced ESCC progression

**Figure 7:**
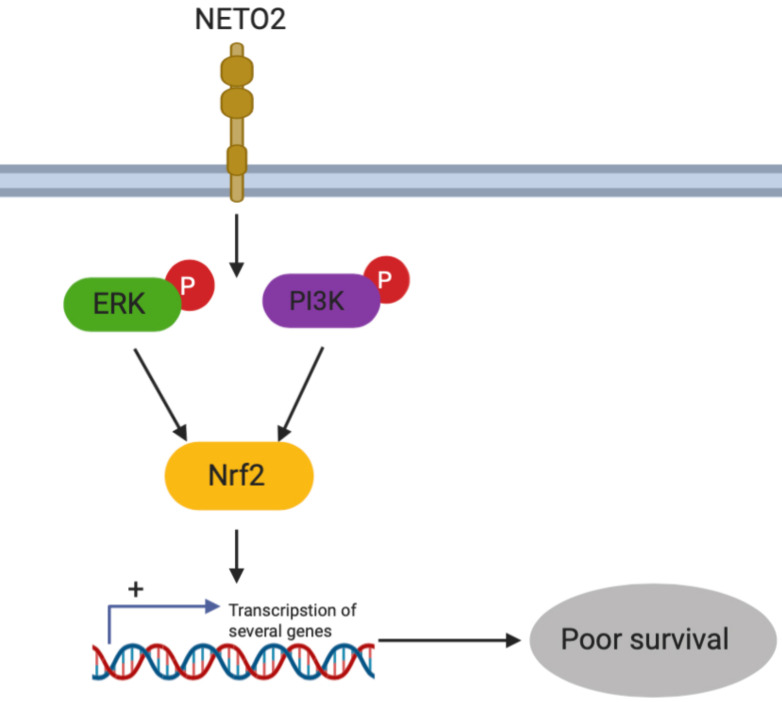
Schematic diagram summarizing the NETO2-induced ERK/PI3K pathway.

Nrf2 was found to play a role in lots of cancers by promoting the cancer cell proliferation and drug resistance. Recent studies have suggested that ERK and AKT activated Nrf2 expression 19,20. Interestingly, our results indicated that Nrf2 was dramatically downregulated by NETO2 knockdown (Figure 6F). ESCC cell proliferation was also prominently inhibited by Nrf2-siRNA treatment, and this inhibitory effect was much lower in ECA109 cell lines (Figure 6G). We also investigated EMT biomarkers expression after knockdown Nrf2 (Figure 6I). Interestingly, in the hypoxic microenvironment, knockdown of Nrf2 led to an obvious effect on cell migration and invasion compared with basal conditions (Figure 6H,J). The discrepancy may be attributed to the function of Nrf2 in oxidative stress through regulating transcriptional genes in anti-apoptotic environments and tumorigenesis. Depletion of Nrf2 also downregulated snail and MMP2 protein levels, but upregulated E-cadherin. These results indicate that Nrf2 might be downstream of NETO2 and could lead to a novel therapeutic target for the treatment of ESCC (Figure 7).

## Discussion

Although the progress made in elucidating its molecular mechanisms, ESCC is continues to have high mortality rates, high probabilities of metastasis, and poor prognoses. Surgery has been the primary curative treatment of ESCC because of complex tumorigenesis progression and paucity of targeted therapies. Previous studies of NETO2 have implicated this gene in neuron-specific processes such as brain excitatory synaptic conduction 9,10. Recently studies have also linked NETO2 to an oncogene in various types of cancers. These studies focusing on renal and colorectal carcinoma demonstrated that measurement of NETO2 expression could potentially serve as a tool for early diagnosis and for prediction of advanced tumor progression 16,21. NETO2 was found closely combined with clinicopathological features and promoted metastasis in gastric cancer 22. However, the role and clinical relevance of NETO2 are poorly understood in ESCC.

In the present study, our research revealed that NETO2 plays significant roles in ESCC progression. NETO2 expression was markedly elevated in ESCC as compared with adjacent control tissues. High levels of NETO2 were also a marker of poor survival in ESCC patients. Additionally, our experiments observed that NETO2 was a key regulator that had crucial roles in ESCC cell proliferation, antagonizing apoptosis, and EMT. We then measured cell apoptosis-related signaling molecules and the data demonstrated that NETO2 antagonized apoptosis by regulating the intrinsic apoptotic pathway. NETO2 overexpression or knockdown altered the caspase-dependent apoptosis pathway through regulating the expression of caspase-3, PARP and Bcl-2 protein family members. In response to apoptotic stimuli, as we previously described 23, cytochrome c is released from the mitochondria because of mitochondrial outer membrane permeabilization (MMOP), which is mediated by Bcl-2 and related proteins and triggers the other caspase cascade that leads to apoptosis.

Metastasis is a complex cellular process. At the molecular level, proteolytic degradation of ECM and EMT are two critical steps. EMT is central in tumor metastasis and resistance to therapy 24. In this process, tumor cells lose the epithelial potential and convert to a mesenchymal morphology, which is accompanied by increased expression of mesenchymal markers and decreased expression of epithelial markers 24,25. In our experiments, overexpression NETO2 not only resulted in EMT morphological changes but also in decreased expression of epithelial-related markers (such as E-cadherin, which was a key component of adherens junctions and cell-to-cell adhesive progress). In contrast, NETO2 depletion increased expression of E-cadherin and downregulated mesenchymal markers such as vimentin, and N-cadherin 26. In our work, we also observed that knockdown of NETO2 altered snail, slug and MMP2 protein expression. Mechanically, during EMT, cooperation among different transcription factors such as Snail, Slug, ZEB1/2 and Twist result in the orchestration of the Cadherin switches responsible for the changes in the adhesion properties of the EMT cells promoting mesenchymal morphology 27. Previous reports have shown that ESCC tumor invasion depth and lymph node metastasis are related to MMP-2 28,29. Herein, our data demonstrated that NETO2 promoted ESCC cells migration via modulating EMT related markers.

According to previous research, NETO2 could activate PI3K/AKT axis, thereby promoting GC cell EMT 22. It is well known that the MEK/ERK pathway and PI3K/AKT pathways have been implicated in tumorigenic roles in a variety of cancers. In our works, we found that NETO2 could regulate the phosphorylation proteins and promote cell proliferation and highly malignant phenotype in ESCC via the PI3K/AKT and ERK pathways concomitantly. Nrf2, also known as nuclear factor E2-related factor 2, a member of the NF-E2 family, is a cap 'n' collar basic leucine-zipper transcription factor 30. It assimilates cellular signals and responds by directing multiple transcriptional factors that protect cells from oxidative insult and maintain cellular redox homeostasis. Recently, more and more analyses have indicated that Nrf2 can promote oncogenesis 31. Genetic analyses of human tumors have shown that stimulation of Nrf2 may conversely be oncogenic and cause resistance to chemotherapy 32,33. However, the precise mechanisms remain unknown. In our study, Nrf2 was found to play a potential role in ESCC tumor progression. Interestingly, in the hypoxic microenvironment, knockdown of Nrf2 led to obvious effects on cell migration and invasion compared with basal conditions. This discrepancy may be attributed to the function of Nrf2 in oxidative stress though regulating transcriptional genes in anti-apoptotic and tumorigenesis. What's more, a recent study suggested that ERK and AKT activated Nrf2 expression, and Nrf2 might be downstream of Neto2-mediated ERK and PI3K pathway 19,20. Taken together, our results revealed that Nrf2 is a critical downstream event responsible for NETO2-mediated PI3K/Akt and ERK signaling pathways and plays a crucial role in ESCC cell progression. However, further studies are needed to confirm the precisely role of Nrf2 in these signaling pathways.

In conclusion, although the current markers of proliferation and metastasis was used in ESCC, alterations of drug targets are infrequent in patients. Our present study demonstrated that NETO2 is a novel marker which highly associated with clinicopathologic features in ESCC patients. Furthermore, downregulation of NETO2 reduced proliferation and metastasis capability via ERK and PI3K/AKT pathways by regulating Nrf2 expression (Figure 7). NETO2 could be a potential therapy approach and novel prognostic biomarker in ESCC patients.

## Figures and Tables

**Table 1:**
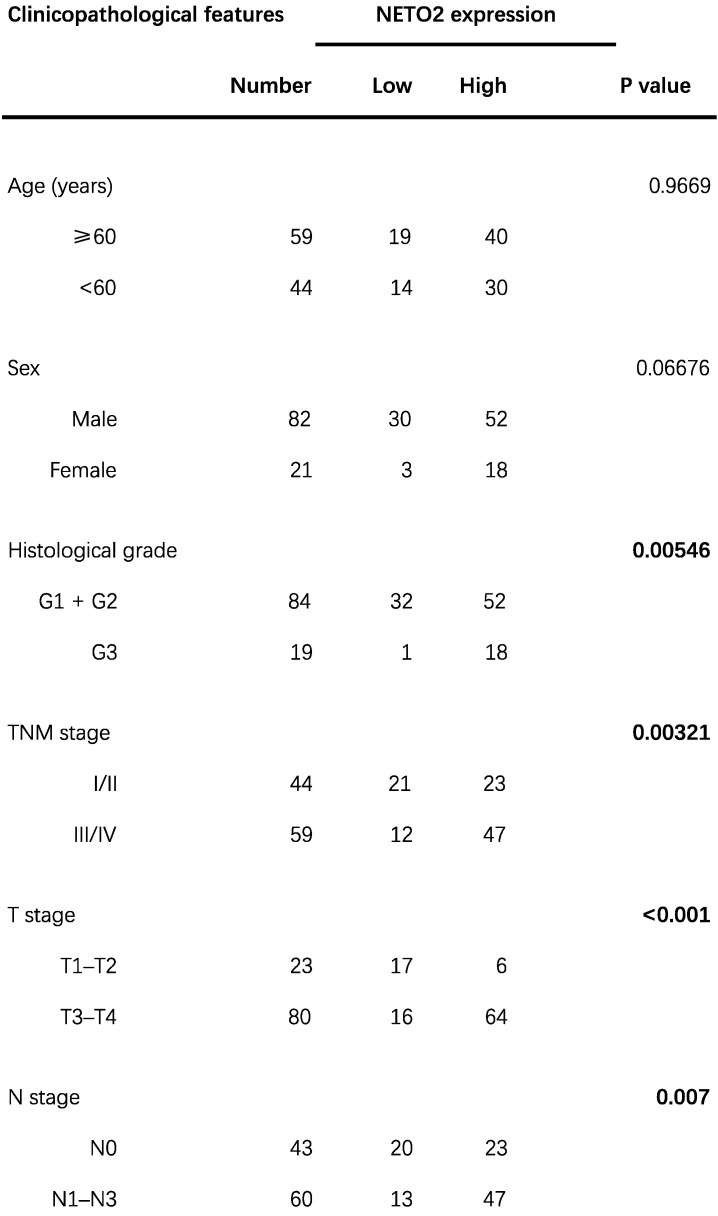
Association of NETO2 expression with clinicopathological characteristics of ESCC

## References

[1] Chen W, Zheng R, Baade PD, Zhang S, Zeng H, Bray F (2016). Cancer statistics in china, 2015. CA Cancer J Clin.

[2] Rustgi AK, El-Serag HB (2014). Esophageal carcinoma. N Engl J Med.

[3] Liu L, Lin C, Liang W, Wu S, Liu A, Wu J (2015). Tbl1xr1 promotes lymphangiogenesis and lymphatic metastasis in esophageal squamous cell carcinoma. Gut.

[4] Roth JA, Putnam JB Jr (1994). Surgery for cancer of the esophagus. Semin Oncol.

[5] Flanagan FL, Dehdashti F, Siegel BA, Trask DD, Sundaresan SR, Patterson GA (1997). Staging of esophageal cancer with 18f-fluorodeoxyglucose positron emission tomography. AJR Am J Roentgenol.

[6] Hosch SB, Stoecklein NH, Pichlmeier U, Rehders A, Scheunemann P, Niendorf A (2001). Esophageal cancer: The mode of lymphatic tumor cell spread and its prognostic significance. J Clin Oncol.

[7] Liang C, Shi S, Qin Y, Meng Q, Hua J, Hu Q (2020). Localisation of pgk1 determines metabolic phenotype to balance metastasis and proliferation in patients with smad4-negative pancreatic cancer. Gut.

[8] Zhao Y, Wei L, Shao M, Huang X, Chang J, Zheng J (2017). Brca1-associated protein increases invasiveness of esophageal squamous cell carcinoma. Gastroenterology.

[9] Stohr H, Berger C, Frohlich S, Weber BH (2002). A novel gene encoding a putative transmembrane protein with two extracellular cub domains and a low-density lipoprotein class a module: Isolation of alternatively spliced isoforms in retina and brain. Gene.

[10] Zhang W, St-Gelais F, Grabner CP, Trinidad JC, Sumioka A, Morimoto-Tomita M (2009). A transmembrane accessory subunit that modulates kainate-type glutamate receptors. Neuron.

[11] Straub C, Zhang W, Howe JR (2011). Neto2 modulation of kainate receptors with different subunit compositions. J Neurosci.

[12] Pressey JC, Mahadevan V, Khademullah CS, Dargaei Z, Chevrier J, Ye W (2017). A kainate receptor subunit promotes the recycling of the neuron-specific k(+)-cl(-) co-transporter kcc2 in hippocampal neurons. J Biol Chem.

[13] Ivakine EA, Acton BA, Mahadevan V, Ormond J, Tang M, Pressey JC (2013). Neto2 is a kcc2 interacting protein required for neuronal cl- regulation in hippocampal neurons. Proc Natl Acad Sci U S A.

[14] Li Y, Zhang Y, Liu J (2019). Neto2 promotes pancreatic cancer cell proliferation, invasion and migration via activation of the stat3 signaling pathway. Cancer Manag Res.

[15] He AR, Zhu Q, Gao S (2019). Reducing neto2 expression prevents human nasopharyngeal carcinoma (npc) progression by suppressing metastasis and inducing apoptosis. Biochem Biophys Res Commun.

[16] Hu L, Chen HY, Cai J, Yang GZ, Feng D, Zhai YX (2015). Upregulation of neto2 expression correlates with tumor progression and poor prognosis in colorectal carcinoma. BMC Cancer.

[17] Villa E, Critelli R, Lei B, Marzocchi G, Camma C, Giannelli G (2016). Neoangiogenesis-related genes are hallmarks of fast-growing hepatocellular carcinomas and worst survival. Results from a prospective study. Gut.

[18] Li M, Zhang Z, Li X, Ye J, Wu X, Tan Z (2014). Whole-exome and targeted gene sequencing of gallbladder carcinoma identifies recurrent mutations in the erbb pathway. Nat Genet.

[19] Yamadori T, Ishii Y, Homma S, Morishima Y, Kurishima K, Itoh K (2012). Molecular mechanisms for the regulation of nrf2-mediated cell proliferation in non-small-cell lung cancers. Oncogene.

[20] Kim KC, Kang KA, Zhang R, Piao MJ, Kim GY, Kang MY (2010). Up-regulation of nrf2-mediated heme oxygenase-1 expression by eckol, a phlorotannin compound, through activation of erk and pi3k/akt. Int J Biochem Cell Biol.

[21] Snezhkina AV, Nyushko KM, Zaretsky AR, Shagin DA, Sadritdinova AF, Fedorova MS (2018). [transcription factor sap30 is involved in the activation of neto2 gene expression in clear cell renal cell carcinoma]. Mol Biol (Mosk).

[22] Liu JY, Jiang L, He T, Liu JJ, Fan JY, Xu XH (2019). Neto2 promotes invasion and metastasis of gastric cancer cells via activation of pi3k/akt/nf-kappab/snail axis and predicts outcome of the patients. Cell Death Dis.

[23] Xu JC, Zhou XP, Wang XA, Xu MD, Chen T, Chen TY (2019). Cordycepin induces apoptosis and g2/m phase arrest through the erk pathways in esophageal cancer cells. J Cancer.

[24] Pastushenko I, Brisebarre A, Sifrim A, Fioramonti M, Revenco T, Boumahdi S (2018). Identification of the tumour transition states occurring during emt. Nature.

[25] Ye X, Weinberg RA (2015). Epithelial-mesenchymal plasticity: A central regulator of cancer progression. Trends Cell Biol.

[26] Shenoy AK, Jin Y, Luo H, Tang M, Pampo C, Shao R (2016). Epithelial-to-mesenchymal transition confers pericyte properties on cancer cells. J Clin Invest.

[27] Gugnoni M, Sancisi V, Manzotti G, Gandolfi G, Ciarrocchi A (2016). Autophagy and epithelial-mesenchymal transition: An intricate interplay in cancer. Cell Death Dis.

[28] Shay G, Lynch CC, Fingleton B (2015). Moving targets: Emerging roles for mmps in cancer progression and metastasis. Matrix Biol.

[29] Wang X, Lu N, Niu B, Chen X, Xie J, Cheng N (2012). Overexpression of aurora-a enhances invasion and matrix metalloproteinase-2 expression in esophageal squamous cell carcinoma cells. Mol Cancer Res.

[30] McMahon M, Itoh K, Yamamoto M, Chanas SA, Henderson CJ, McLellan LI (2001). The cap'n'collar basic leucine zipper transcription factor nrf2 (nf-e2 p45-related factor 2) controls both constitutive and inducible expression of intestinal detoxification and glutathione biosynthetic enzymes. Cancer research.

[31] Sporn MB, Liby KT (2012). Nrf2 and cancer: The good, the bad and the importance of context. Nat Rev Cancer.

[32] Shibata T, Kokubu A, Gotoh M, Ojima H, Ohta T, Yamamoto M (2008). Genetic alteration of keap1 confers constitutive nrf2 activation and resistance to chemotherapy in gallbladder cancer. Gastroenterology.

[33] Homma S, Ishii Y, Morishima Y, Yamadori T, Matsuno Y, Haraguchi N (2009). Nrf2 enhances cell proliferation and resistance to anticancer drugs in human lung cancer. Clin Cancer Res.

